# Blocking abdominal lymphatic flow attenuates acute hemorrhagic necrotizing pancreatitis -associated lung injury in rats

**DOI:** 10.1186/1476-9255-10-9

**Published:** 2013-03-12

**Authors:** He Peng, Wang Zhi-fen, Jin Su-mei, Guo Yun-zhen, Li Yan, Chen Li-ping

**Affiliations:** 1 Department of General Surgery, The First Affiliated Hospital of Xinxiang Medical University, Weihui 453100, P. R. China; 2 Department of Endocrinology, The First Affiliated Hospital of Xinxiang Medical University, Weihui 453100, P. R. China; 3 Department of Neurosurgery, The First Affiliated Hospital of Xinxiang Medical University, Weihui 453100, P. R. China

**Keywords:** Thoracic duct ligation, Thoracic duct drainage, Acute hemorrhagic necrotizing pancreatitis, Acute lung injury

## Abstract

**Background:**

Acute hemorrhagic necrotizing pancreatitis (AHNP) is a severe acute inflammatory of pancreas that can lead to extrapancreatic organ disfunction. The lung and intestine is the most common involved organs, and abdominal lymphatic flow may contribute to AHNP- associated organ injury. In this study, we investigated the impact of thoracic duct ligation and drainage on lung and intestine injury in rats with AHNP.

**Methods:**

Thirty-two male Wistar rats were randomly divided into 4 groups: sham operation group, AHNP group, AHNP + ligation group and AHNP + drainage group. Rat AHNP model was induced by retrograde injection of 3.5% sodium deoxycholate into the biliopancreatic duct, and the sham operation group was injected only with saline. In AHNP + ligation group and AHNP + drainage group, thoracic duct was ligated or drainaged before model induction. At 6 h after model induction, the bronchoalveolar lavage fluid (BALF) were collected for determination of tumor necrosis factor-alpha (TNF-alpha), and the tissues of lung, intestine and pancrease were harvested individually for pathohistological evaluation and the myeloperoxidase (MPO) activity determination. In addition, the activity of serum amylase and diamine oxidase (DAO) was determined in each group.

**Results:**

The pathohistological damage and MPO of lung, intestine and pancrease, TNF-alpha of BALF, serum amylase and DAO were all increased in AHNP group compared to those in sham operation group (P < 0.05). In AHNP + ligation group, the pathohistological damage and MPO of lung and TNF-alpha of BALF were reduced, but the pathohistological damage and MPO of intestine and pancrease were increased compared with AHNP group (P < 0.05), however the activity of serum amylase and DAO was no changed. In AHNP + drainage group, the pathohistological damage and MPO of lung, intestine and pancrease, TNF-alpha of BALF, serum amylase and DAO were all reduced compared to those in AHNP group (P < 0.05).

**Conclusions:**

Our finding suggest that thoracic duct ligation can reduce neutrophil infiltration and TNF-alpha release and then attenuates lung injury in rats with AHNP, but aggravates the injury of intestine and pancrease. While thoracic duct drainage attenuates the injury of lung, intestine and pancreaseat the same time in rats with AHNP.

## Background

Acute hemorrhagic necrotizing pancreatitis (AHNP) is a severe acute inflammatory disease of the pancrease that can lead to intestinal barrier failure and subsequent translocation of bacterial and endotoxin, which promotes the development of multiple organ dysfunction. The lung is the most common involved extra-gut organ both in AHNP patients and animal model [1,2]. But the mechanism uderlying intestinal barrier failure and lung injury induced by AHNP is still not clear.

We have known that abdominal have rich lymphatic vessels and drainage system, and the permeability of lymphatic capillary is much higher than that of capillary, so more easily the bacteria and endotoxin enter the lymphatic capillary, through thoracic lymph duct and thoracic lymph duct to the left jugular vein, and then back to the right atrium, distribute to the lungs with pulmonary artery. So in a certain sense the lung is the first organ accepting and bearing the reflux of intestine lymphatic involved those inflammatory substances such as bacterial, endotoxin, cytokine, etc. In AHNP patients, when thoracic duct was drained, beneficial effects were seen in some, but not all of them [3-5]. In AHNP animals, when thoracic lymphatic duct was ligated, the morbidity of rat was decreased in one study [6]. In another study, however, the same procedure induced pancreatic edema and pancreatitis in rat [7]. From those, how lymph-flow interruption effects AHNP remains a question.

This study was undertaken to revisit the question in the rat. We hypothesized that abdominal lymphatic system is a way of inflammatory substances from intestine translocate to the lung, and investigated the impact of interruption of abdominal lymphatic flow on the injury of lung, intestine and pancrease in rats with AHNP by thoracic duct ligation and drainage.

## Materials and methods

### Animals

Adult male wistar rats weighing 230 to 250 g were provided by the Chinese Academy of Medical Sciences Institute of Radiation Laboratory Animal Center, Beijng, China. The animals were kept in a 12 h light/dark cycle with free access to water and a standard chow. After a 7-day acclimation, they were randomly divided into 4 groups, namely sham operation group, AHNP group, AHNP + ligation group and AHNP + drainage group. All experiments were performed in accordance with the animal care and handling guidelines of the Committee on Animal Care of Tianjin. Experimental protocol was carried out according to the time schedule (Table 1).

**Table 1:** Time schedule

**Group**	**−1 ~ 0 h**	**0 h**	**6 h**
sham operation	expose thoracic duct	sham operation	sacrifice
AHNP	expose thoracic duct	model induction	sacrifice
AHNP + ligation	ligate thoracic duct	model induction	sacrifice
AHNP + drainage	drainage thoracic duct	model induction	sacrifice

### Thoracic duct lagation and drainage

All rats were intra-abdominally anesthetized by chloral hydrate (300 mg/kg). A midline incision was made to open abdominal cavity. After a medial viceral rotation, the part of parietal peritoneum covering the left kidney was exposed. A small incision was made on it. Thoracic duct was located to the left of abdominal aorta. Using #0 silk suture, we ligated thoracic duct near the diaphragm side in AHNP + ligation group. As in AHNP + drainage group, after thoracic duct lagation, we drainaged thoracic duct at the place where thoracic duct started. Laparotomy was performed and thoracic duct was exposed only in sham operation group and AHNP group. Finally, intestine was put in their original places.

### Induction of AHNP

After thoracic duct lagation or drainage, the common bile duct was clamped in the hepatoduodenal ligament by a small bulldog clamp, and the biliopancreatic duct was cannulated through mammary papilla from the anterior wall of the duodenum. 3.5% sodium taurocholate (Sigma, St. Louis, MO, USA) was injected by the cannula with an even speed of 0.15 mL/min (1 m L/k g body weight) using syringe pump (LP, FA343, Beijing, China), and the atraumatic vascular clamp was removed 10 min later. The sham operation group were injected only with saline in the same way. Finally, the abdominal incision were closed in two layers. All procedures were performed using a sterile technique.

### Bronchoalveolar lavage and experimental sample obtained

At six hours after injection of sodium deoxycholate, blood samples were drawn from the aorta, centrifuged at 400 g for 10 min, and kept at −20°C for determination of serum amylase, diamine oxdase (DAO) and tumor necrosis factor-α (TNF-α).

Then all animals were killed by aortic exsanguination, the lung was carefully dissected to the level of the carina after removal of the heart. The left main stem bronchus was tied proximally and removed. The right lung was perfused in situ immediately by intratracheal infusion of three 3-mL aliquots of PBS (pH 7.4). The lavage fluid was kept in the lungs for 3 min and recovered by gravity into tubes on ice. The recovery was 7.5 mL of total 9 mL. The bronchoalveolar lavage fluid (BALF) was centrifuged at 400 g for 10 min, and the supernatant was kept at −20°C for analyzing of TNF-α. Finally tissues of left lung, intestine (3–5 cm from the ileocecal valve) and pancrease were harvested individually and then were fixed in 5% formaldehyde for pathohistological evaluation or saved at −70°C for determination the activity of myeloperoxidase (MPO) and DAO.

### Histologic examination and grading

Tissue samples of lung, intestine and pancrease were embedded in paraffin and cut in 5 μm-thick sections. After de-paraffinization, sections were stained with hematoxylin and eosin stain and examined by two investigators in a blinded manner. The specimens of lung and pancrease were scored according to the criteria described previously [8,9]. The intestinal mucosal injury score was graded on a six-tiered scale defined by Chiu [10].

### MPO activity determination

MPO activities were determined using an MPO kit produced by Jiancheng Bioengineering Institute (Nanjing, China) according to the manufacturer’s instruction. In brief, frozen samples of lung, intestine and pancrease were thawed and homongenized in ice-cold buffer provided in the kit. The homogenates were centrifuged at 5000 g for 10 min. Pellets were suspended in 0.5% hexadecyl trimethyl ammonium bromide in 50 mM PBS (pH 6.0) and incubated at 60°C for 2 hours. After another centrifugation, supernatants were collected. Their protein concentrations were measured using a protein assay kit (A045, Jiancheng Bioengineering Institute). In a 96-well plate, 15 μg protein was incubated with 100 μl 3,3^′^,5,5^′^-tetramethylbenzidine for 3 min. After 100 μl sulphuric acid (1 N) was added, absorbance was read in a spectrophotometer using a wavelength of 450 nm. Original MPO value was normalized with protein contents.

### Analysis of serum amylase and TNF-α in lung-lavage fluids and serum

Serum amylase was measured with automatic biochemical analyzer. TNF-α in serum and lung-lavage fluids were determined using ELISA kits (RayBiotech, Guangzhou, China) according to manufacturer protocol.

### Determination of the activity of DAO in serum and intestine

The activity of DAO in serum and intestine was determined using enzymatic spectrophotometry, as described previously [11]. Briefly, 0.5 mL serum was mixed with a solution containing 0.1 mol/L PBS (3 mL, pH 7.2), horseradish peroxidase (4 μg, 0.1 mL), 3,3^′^-dimethoxybenzidine (500 μg, 0.1 mL) and cadaverine dihydrochloride (175 μg, 0.1 mL) and incubated at 37°C for 30 min. The OD_436_ was detected and the DAO activity was measured. About 0.5 mL intestinal homogenates was used to measure the DAO activity with the same method.

### Statistics

The data were analyzed using SPSS PC version 11.5. Results were expressed as mean ± SD. Data were analyzed by one-way ANOVA analysis of variance and a *P* value < 0.05 was considered significant.

## Results

### The effect of blocking abdominal lymphatic flow on histopathological injury of lung, intestine and pancrease in AHNP rats

#### Lung

**Figure 1:**
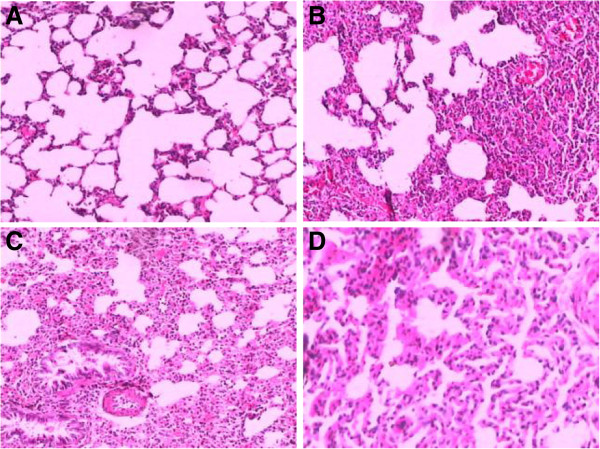
**The effect of blocking abdominal lymphatic flow on histopathological change of lung in AHNP rats. A**: Lung in a sham operation animal 6 hr after injection of saline, mild alveolar septum edema and inflammatory infiltrate. **B**: AHNP group, diffuse pulmonary capillary dilatation and congestion, intense alveolar septum swelling and breakage, part of alveolar structural damage and heavy neutrophils infiltration. **C** and **D**: AHNP + ligation and AHNP + drainage group, moderate to severe alveolar septum swelling and neutrophils infiltration (H & E, × 100).

Diffuse pulmonary capillary dilatation and congestion, intense alveolar septum swelling and breakage, part of alveolar structural damage and heavy infiltration of inflammatory cells mostly neutrophils were found in the lung tissue of AHNP group (Figure 1B). The mean histopathologic scores of AHNP group were higher than those in sham operation group (*P* < 0.05, Table 2). In AHNP + ligation and AHNP + drainage group, the major histopathological findings were moderate to severe edema of the alveolar walls and alveolar blood stasis with moderate infiltration of neutrophils (Figure 1C, D), and the mean histopathologic scores were all decreased significantly compared to those of AHNP group (P < 0.05, Table 2).

**Table 2:** The effect of blocking abdominal lymphatic flow on histopathological injury of lung, intestine and pancrease in AHNP rats (mean ± SD)

**Group**	**n**	** Histopathologic scores**		
		**Lung**	**Intestine**	**Pancrease**
Sham operation	8	0.86 ± 0.31	0.68 ± 0.21	1.07 ± 0.21
AHNP	8	2.57 ± 0.49^*^	4.15 ± 0.70^*^	6.27 ± 1.04^*^
AHNP + ligation	8	2.05 ± 0.27^*#^	4.67 ± 0.61^*^	6.18 ± 1.09^*^
AHNP + drainage	8	1.99 ± 0.28^*#^	2.90 ± 0.69^*#a^	4.82 ± 1.03^*#a^

^*^P < 0.05 compared with sham group.

^#^*P* < 0.05 compared with AHNP group.

^a^*P* < 0.05 compared with AHNP + ligation group.

#### Intestine

**Figure 2:**
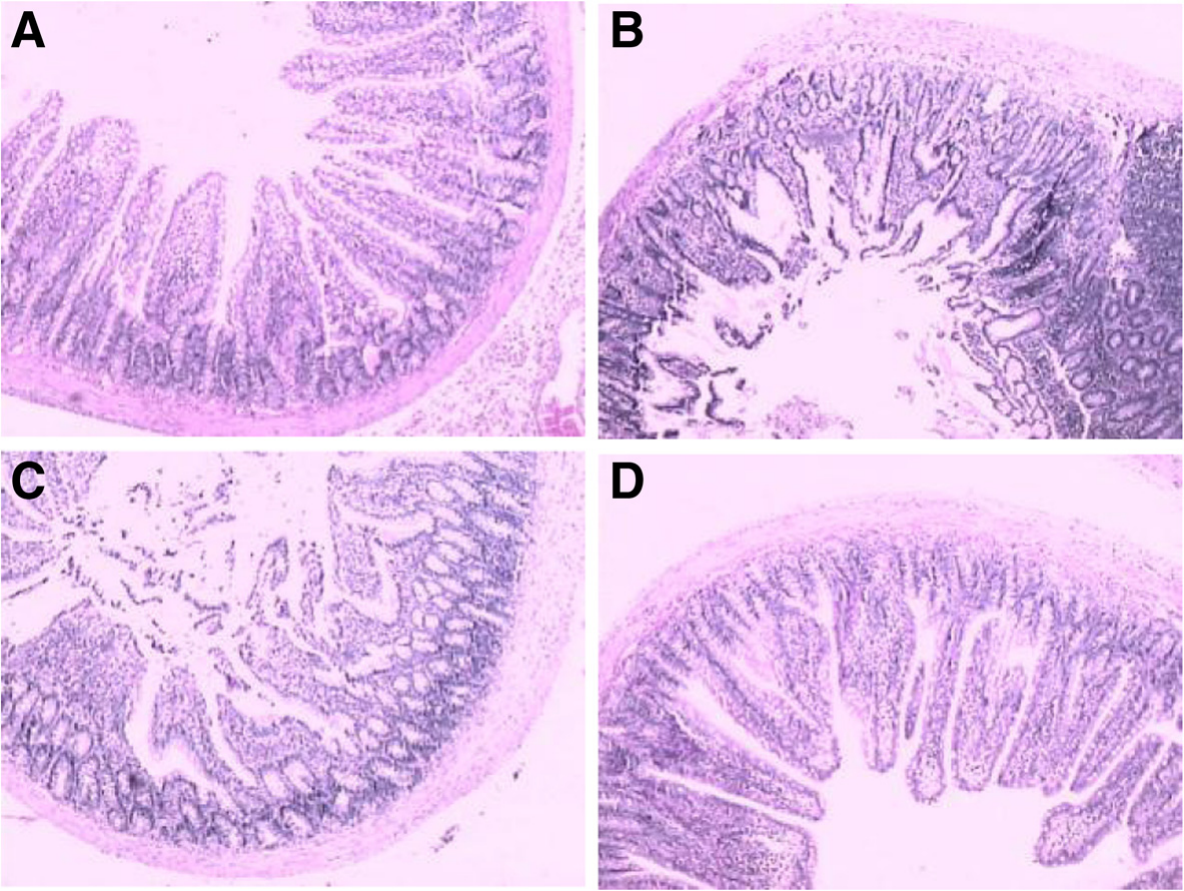
**The effect of blocking abdominal lymphatic flow on histopathological change of intestine in AHNP rats. A**: intestine in a sham operation, normal intestinal mucosa. **B** and **C**: AHNP group and AHNP + ligation group, focal necrosis of intestinal mucosa, inflammatory cell infiltration in various mucosa layers and exuviations of microvillus top of epithelium. **D**: AHNP + drainage group, villi with patchy disruption of the epithelial cells, mild congestion edema and inflammatory cell infiltration (H & E, × 100).

Congestion edema and focal necrosis of intestinal mucosa, inflammatory cell infiltration in various mucosa layers, dilation of central chyle vessel and exuviations of microvillus top of epithelium of intestinal mucosa were found in AHNP group (Figure 2B). And the mean histopathologic scores of AHNP group were significantly higher than those in sham operation group (*P* < 0.01, Table 2). The histopathological injury and scores of intestine in AHNP + ligation group were similar to those in AHNP group (Figure 2C). But in AHNP + drainage group, the major histopathological findings were mild congestion edema and inflammatory cell infiltration of intestinal mucosa without necrosis, and mild exuviations of microvillus top of epithelium of intestinal mucosa (Figure 2D), and the the mean histopathologic scores of AHNP + drainage group was decreased significantly compared to those of AHNP group or AHNP + ligation group (*P* < 0.05).

#### Pancrease

**Figure 3:**
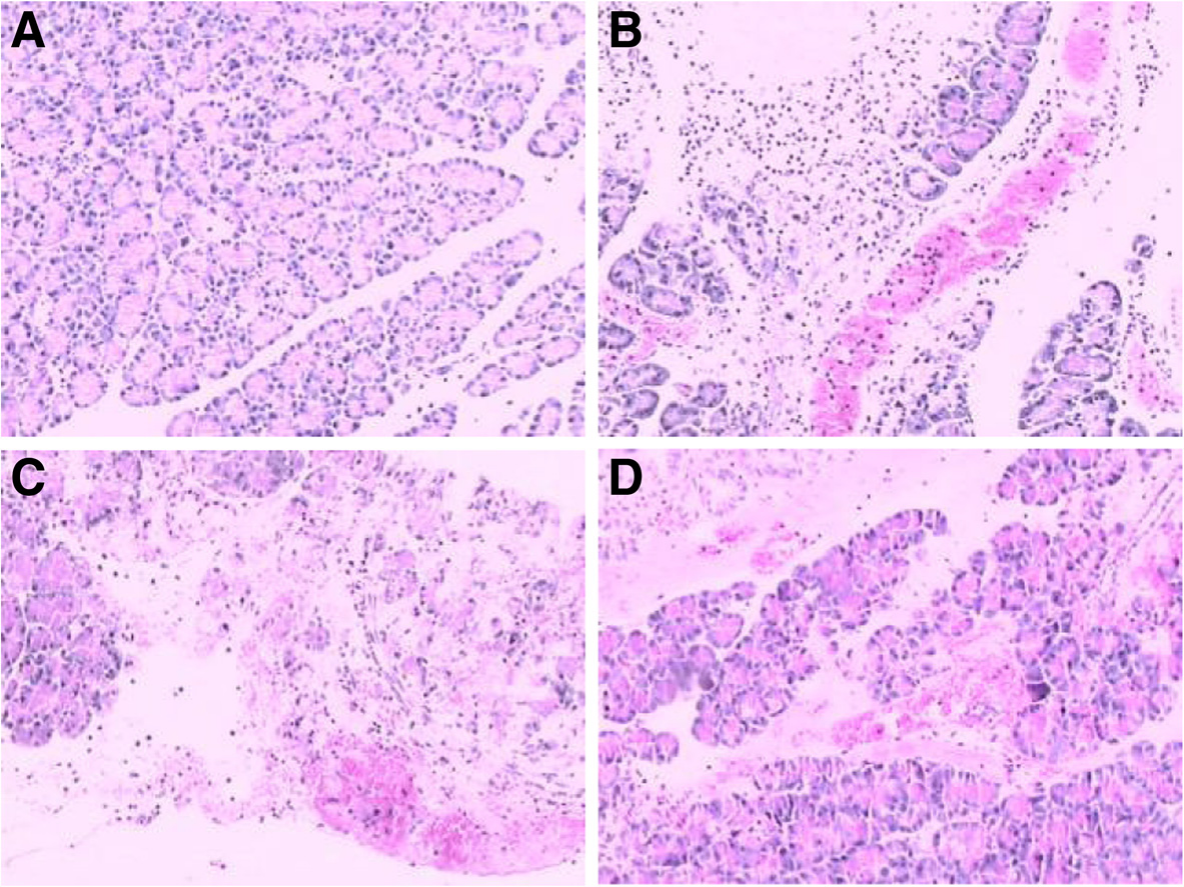
**The effect of blocking abdominal lymphatic flow on histopathological change of pancrease in AHNP rats. A**: pancrease in a sham operation animal, the glandular architecture is almost normal. **B** and **C**: AHNP group and AHNP + ligation group, extensive necrosis of pancreatic tissue, intense hemorrhage and inflammatory infiltrate. **D**: AHNP + drainage group, localized necrosis of pancreatic tissue and moderate hemorrhage (H & E, × 100).

Extensive necrosis of pancreatic tissue, intense hemorrhage and inflammatory infiltrate were found in AHNP group. The histopathological injury of pancrease in AHNP + ligation group were similar to those in AHNP group (Figure 3B), but in AHNP + drainage group (Figure 3C), but the necrosis of pancreatic tissue and hemorrhage were mitigated compare to those in AHNP group (Figure 3D). The sham operation group was normal (Figure 3A). The mean histopathologic scores of pancrease in all groups were shown in Table 2.

### The effect of blocking abdominal lymphatic flow on MPO activity of lung, intestine and pancrease in AHNP rats

The MPO levels of lung, intestine and pancrease in AHNP group were significantly higher than those in sham operation group. In AHNP + ligation group, the lung MPO level were decreased, but the MPO levels of intestine and pancrease were increased compare to those in AHNP group. However the MPO levels of lung, intestine and pancrease were all decreased in AHNP + drainage group (Table 3).

**Table 3:** The effect of blocking abdominal lymphatic flow on MPO activity of lung, intestine and pancrease in AHNP rats (U/g, mean ± SD)

**Group**	**n**	** MPO**		
		**Lung**	**Intestine**	**Pancrease**
sham operation	8	0.71 ± 0.14	0.66 ± 0.14	0.64 ± 0.13
AHNP	8	1.24 ± 0.35^*^	1.17 ± 0.25^*^	1.25 ± 0.27^*^
AHNP + ligation	8	0.86 ± 0.24^#^	1.72 ± 0.50^*#^	1.70 ± 0.27^*#^
AHNP + drainage	8	0.88 ± 0.23^#^	0.90 ± 0.18^a^	0.86 ± 0.14^#a^

^*^P < 0.05 compared with sham group.

^#^*P* < 0.05 compared with AHNP group.

^a^*P* < 0.05 compared with AHNP + ligation group.

### The effect of blocking abdominal lymphatic flow on serum amylase and TNF-α in AHNP rats

Serum amylase and TNF-α were increased significantly in AHNP group compared to that in sham operation group. There were no significant difference between AHNP + ligation group and AHNP group, but in AHNP + drainage group, serum amylase and TNF-α were decreased compared to that in AHNP group (Table 4).

**Table 4:** The effect of blocking abdominal lymphatic flow on serum amylase and TNF-α in AHNP rats (mean ± SD)

**Group**	**n**	**Amylase (U/L)**	**TNF-α (μg/L)**
sham operation	8	3078.8 ± 526.5	0.62 ± 0.08
AHNP	8	8748.5 ± 1097.8^*^	2.07 ± 0.43^*^
AHNP + ligation	8	8988.8 ± 1022.8^*^	1.89 ± 0.46^*^
AHNP + drainage	8	5229.3 ± 1327.7^*#a^	0.91 ± 0.20^#a^

^*^P < 0.05 compared with sham group.

^#^*P* < 0.05 compared with AHNP group.

^a^*P* < 0.05 compared with AHNP + ligation group.

### The effect of blocking abdominal lymphatic flow on protein and TNF-α of BALF in AHNP rats

TNF-α in BALF were increased significantly in AHNP compared to that in sham operation group. But blocking abdominal lymphatic flow (thoracic duct ligation or drainage) could significantly reduce the level of TNF-α of BALF in AHNP rats (Table 5).

**Table 5:** The effect of blocking abdominal lymphatic flow on protein and TNF-α in BALF in AHNP rats (mean ± SD)

**Group**	**n**	**Protein (g/L)**	**TNF-α (μg/g pro)**
sham operation	8	0.21 ± 0.05	3.30 ± 0.97
AHNP	8	0.62 ± 0.11 ^*^	4.79 ± 1.16^*^
AHNP + ligation	8	0.24 ± 0.07 ^#^	3.27 ± 1.07^#^
AHNP + drainage	8	0.22 ± 0.06 ^#^	3.18 ± 1.02^#^

^*^P < 0.05 compared with sham group.

^#^*P* < 0.05 compared with AHNP group.

### The effect of blocking abdominal lymphatic flow on DAO activity in serum and intestine in AHNP rats

Serum DAO activity was increased and intestine DAO activity was decreased significantly in AHNP and AHNP + ligation group compared to that in sham operation group. But thoracic duct drainage could significantly reduce serum DAO and increase intestine DAO in AHNP rats (Table 6).

**Table 6:** The effect of blocking abdominal lymphatic flow on DAO activity in serum and intestine in AHNP rats (mean ± SD)

**Group**	**n**	** DAO**	
		**Serum (U/L)**	**Intestine (U/g pro)**
sham operation	8	993.7 ± 390.8	553.6 ± 129.2
AHNP	8	1770.1 ± 749.1^*^	344.5 ± 179.1^*^
AHNP + ligation	8	1867.6 ± 897.7^*^	337.9 ± 134.7^*^
AHNP + drainage	8	1110.0 ± 316.9^#a^	495.5 ± 140.7^#a^

^*^P < 0.05 compared with sham group.

^#^*P* < 0.05 compared with AHNP group.

^a^*P* < 0.05 compared with AHNP + ligation group.

## Discussion

Lung injury and intestinal damage are common complications of AHNP, but the pathway that link pancrease, intestine and pulmonary are not fully understood. Portal vein and mesenteric lymph maybe two main potential ways related to the injury of intestinal and lung of AHNP. Previous studies have found that hepatic keffer cell blockade could reduce lung injury in rat with AHNP [12], and liver reticuloendothelial system could reduce metastasis of toxic substances via portal vein. In the other hand, some factors that promote lung injury have been found in gut and mesenteric lymphatics [6,13], mesenteric lymphatics can carry gut-derived factors that contribute to lung injury in hemorrhagic shock models [14,15]. So we speculate that it is lymphatic system, not portal vein who contrabute to the lung injury in the early stage of AHNP. In the present study, we observed the impact of thoracic duct ligation and drainage on lung and intestine injury in rats with AHNP.

White blood cell count, especially the amount of neutrophils was generally increased in both AHNP patients and animal experiment models [16,17]. The adhesion, aggregation and activation of neutrophils is considered to be the first step in causing tissue and organ damage. Sinece MPO is a marker of the neutrophils infiltration, we observed MPO activity and histopathological change simultaneously in the tissue of pancrease, intestine and lung at 6 hours after retrograde injecting sodium deoxycholate into biliopancreatic duct, and found the MPO activity and pathohistological damage of lung, intestine and pancrease were all increased in rats with AHNP, blocking abdominal lymphatic flow, by ligating and drainaging thoracic duct could all attenuate lung injury by reducing neutrophils infiltration, but had diffirent effect on the injury of intestine and pancrease. When thoracic duct was ligated, the MPO activity and pathohistological damage of intestine and pancrease were not decreased; but when thoracic duct was drainaged, the MPO activity and pathohistological damage of intestine and pancrease were all decreased in rats with AHNP. It means it is thoracic duct drainage not thoracic duct ligation could attenuate the damage of lung, intestine and pancrease at the same time.

DAO is a highly active intracelluar enzyme located in the upper part of intestinal mucosa in human as well as in mammals. DAO activity has been used as an index of small intestinal mucosal mass and integrity [18,19]. When mucosal cells are injured and necrotized, DAO is released into the blood or enters the intestinal tract together with necrotic mucosal cells, thus increasing the serum and intestinal tract DAO level and decreasing the DAO levels in intestinal mucosa [12,20]. In the present study, the activity of serum DAO was markedly higher and the activity of DAO in intestinal mucosa was significantly lower in rats with in AHNP rats with or without thoracic duct ligation, However, the activity of serum DAO was dramatically decreased after treatment with thoracic duct drainage in AHNP rats. Moreover, the activity of DAO in intestinal mucosa was markedly increased. These results suggest that thoracic duct drainage can confer protective effects against intestinal mucosal injury caused by AHNP. Combination of pathohistological change and MPO activity, this study indicates that abdominal lymphatic maybe a source of detrimental factors leading to organ injury and dysfunction. When thoracic duct is ligated, the detrimental factors can not be transported to the lungs, but strandes in the abdomine, so it can reduced the injury of lung, but not intestine and pancrease. When thoracic duct is drainaged, the detrimental lymphatic was drainaged from organism, so attenuates the damage of lung, intestine and pancrease simultaneously. Similar results have been shown in hemorrhagic shock-induced lung injury by mesenteric lymph duct ligation [21,22], but it is rarely reported that the effect of the drainage of mesenteric lymph duct or thoracic duct on organ damage. In addition, thoracic duct drainage also decreased serum amylase activity. It maybe a result of protection of pancreatic acinar in rats with AHNP.

It is well-known that the neutrophils recruitment to the lung is associated with increased permeability of the alveolar-capillary barrie which leads to exudation of protein into the alveolar space [23-25]. TNF-α, as the first endogenous proinflammatory factor a, play an important role in the amplification and the continuation of local and systemic inflammatory response [26,27]. Previous studies have shown that TNF-α is up regulated in lung and serum during this early phase of AHNP in rats which in turn activate a second set of inflammatory factors or cascade, including cytokines, lipid mediators, and reactive oxygen species, as well as other factors associated with initiation of immune tissue migration and infiltration [28]. Similar results were observed in our study. Furthermore, we also found interruption of the thoraci lymph flow (ligation or drainage) could reduce TNF-α release and protein exudation in BALF and attenuate the pathohistological damage of lung in AHNP rats.

In conclusion, blocking abdominal lymphatic flow by ligating and drainaging thoracic duct have diffirent effect on the injury of lung, intestine and pancrease. Thoracic duct ligation attenuates lung injury, but aggravates the injury of intestine and pancrease. While thoracic duct drainage attenuates the injury of lung, intestine and pancrease simultaneously in rats with AHNP. These results indicate that abdominal lymph maybe a source of factors leading to organ injury in AHNP. Recent studies have shown that the lipase-generated free fatty acids are the key components resulting in the cytotoxicity of mesenteric lymph in rats with trauma-hemorrhagic shock and the superior mesenteric artery occlusion [29], but the mechanism needs to be further explained.

## Competing interests

The authors declare that they have no competing interests.

## Authors’ contributions

The author, HP carried out the impact of thoracic duct ligation and drainage on lung and intestine injury in rats with AHNP, participated in the analysis of the data and drafted the manuscript. All authors read and approved the final manuscript.

## References

[B1] Fu Q Cui NQ Characteristics and treatment of organ impairment in early stage of severe acute pancreatitis Chinese Journal of Surgery of Integrated Traditional and Western Medicine 2010 16 151 154

[B2] Lichtenstein A Milani R Jr Fernezlian SM Leme AS Capelozzi VL Martins MA Acute lung injury in two experimental models of acute pancreatitis: infusion of saline or sodium taurocholate into the pancreatic duct Crit Care Med 2000 28 1497 1502 10.1097/00003246-200005000-00040 10834702

[B3] Montravers P Chollet-Martin S Marmuse JP Gougerot-Pocidalo MA Desmonts JM Lymphatic release of cytokines during acute lung injury complicating severe pancreatitis Am J Respir Crit Care Med 1995 152 1527 1533 10.1164/ajrccm.152.5.7582288 7582288

[B4] Dugernier T Reynaert MS Deby-Dupont G Roeseler JJ Carlier M Squifflet JP Prospective evaluation of thoracic-duct drainage in the treatment of respiratory failure complicating severe acute pancreatitis Intensive Care Med 1989 15 372 378 2553789 10.1007/BF00261496

[B5] Stone HH Fabian TC Morris ES Failure of thoracic duct drainage to ameliorate life-threatening physiologic derangements of acute alcoholic pancreatitis South Med J 1983 76 613 614 10.1097/00007611-198305000-00021 6342151

[B6] Zou ZD Zhang ZZ Wang L Wang Y Zheng GH The role of mesenteric lymph in pathogenesis of systemic inflammatory response syndrome and systemic complications following severe acute pancreatitis in rats Zhongguo Wei Zhong Bing Ji Jiu Yi Xue 2010 22 206 209 20398463

[B7] Müller M Putzke HP Siegmund E Dummler W Significance of disturbed lymph flow for the pathogenesis of pancreatitis. I. Ligature of the ductus thoracicus in the rat Exp Pathol 1988 33 95 101 10.1016/S0232-1513(88)80132-4 2456224

[B8] Kyogoku T Manabe T Tobe T Role of ischemia in acute pancreatitis: hemorrhagic shock converts edematous pancreatitis to hemorrhagic pancreatitis in rats Dig Dis Sci 1992 37 1409 1417 10.1007/BF01296012 1380424

[B9] Schmidt J Rattner DW Lewandrowski K Compton CC Mandavilli U Knoefel WT Warshaw AL A better model of acute pancreatitis for evaluating therapy Ann Surg 1992 215 44 56 10.1097/00000658-199201000-00007 1731649 PMC1242369

[B10] Chiu CJ McArdle AH Brown R Scott HJ Gurd FN Intestinal mucosal lesion in low-flow states. I. A morphological, hemodynamic, and metabolic reappraisal Arch Surg 1970 101 478 483 10.1001/archsurg.1970.01340280030009 5457245

[B11] Li JY Yu Y Hao J Jin H Xu HJ Determination of diamine oxdase activity in intestinal tissue and blood using spectrophotometry An ji suan he sheng wu zi yuan 1996 18 28 30

[B12] Gloor B Blinman TA Rigberg DA Todd KE Lane JS Hines OJ Reber HA Kupffer cell blockade reduces hepatic and systemic cytokine levels and lung injury in hemorrhagic pancreatitis in rats Pancreas 2000 21 414 420 10.1097/00006676-200011000-00013 11075997

[B13] Magnotti LJ Xu DZ Lu Q Deitch EA Gut-derived mesenteric lymph: a link between burn and lung injury Arch Surg 1999 134 1333 1340 10.1001/archsurg.134.12.1333 10593331

[B14] Adams CA Jr Sambol JT Xu DZ Lu Q Granger DN Deitch EA Hemorrhagic shock induced up-regulation of P-selectin expression is mediated by factors in mesenteric lymph and blunted by mesenteric lymph duct interruption J Trauma 2001 51 625 631 10.1097/00005373-200110000-00001 11586150

[B15] Dayal SD Haskó G Lu Q Xu DZ Caruso JM Sambol JT Deitch EA Trauma/hemorrhagic shock mesenteric lymph upregulates adhesion molecule expression and IL-6 production in human umbilical vein endothelial cells Shock 2002 17 491 495 10.1097/00024382-200206000-00009 12069186

[B16] Hartman H Abdulla A Awla D Lindkvist B Jeppsson B Thorlacius H Regnér S P-selectin mediates neutrophil rolling and recruitment in acute pancreatitis Br J Surg 2012 99 246 255 10.1002/bjs.7775 22109627

[B17] Abdulla A Awla D Thorlacius H Regnér S Role of neutrophils in the activation of trypsinogen in severe acute pancreatitis J Leukoc Biol 2011 90 5 975 982 10.1189/jlb.0411195 21810937

[B18] Luk GD Bayless TM Baylin SB Plasma postheparin diamine oxidase. Sensitive provocative test for quantitating length of acute intestinal mucosal injury in the rat J Clin Invest 1983 71 1308 1315 10.1172/JCI110881 6406546 PMC436992

[B19] Moriyama K Kouchi Y Morinaga H Irimura K Hayashi T Ohuchida A Goto T Yoshizawa Y Diamine oxidase, a plasma biomarker in rats to GI tract toxicity of oral fluorouracil anti-cancer drugs Toxicology 2006 217 233 239 10.1016/j.tox.2005.09.017 16278042

[B20] Hosoda N Nishi M Nakagawa M Hiramatsu Y Hioki K Yamamot DM Structural and functional alterations in the gut of parenterally or enterally fed rats J Surg Res 1989 47 129 133 10.1016/0022-4804(89)90076-0 2547111

[B21] Sambol JT Xu DZ Adams CA Magnotti LJ Deitch EA Mesenteric lymph duct ligation provides long term protection against hemorrhagic shock-induced lung injury Shock 2000 14 416 419 10.1097/00024382-200014030-00030 11028566

[B22] Deitch EA Gut lymph and lymphatics: a source of factors leading to organ injury and dysfunction Ann N Y Acad Sci 2010 12 E103 E111 20961300 10.1111/j.1749-6632.2010.05713.x

[B23] Guice KS Oldham KT Caty MG Johnson KJ Ward PA Neutrophil-dependent, oxygen-radical mediated lung injury associated with acute pancreatitis Ann Surg 1989 210 740 747 10.1097/00000658-198912000-00008 2589887 PMC1357865

[B24] Sochor M Richter S Schmidt A Hempel S Hopt UT Keck T Inhibition of matrix metalloproteinase-9 with doxycycline reduces pancreatitis-associated lung injury Digestion 2009 80 65 73 10.1159/000212080 19494493

[B25] Yang T Mao YF Liu SQ Hou J Cai ZY Hu JY Ni X Deng XM Zhu XY Protective effects of the free radical scavenger edaravone on acute pancreatitis-associated lung injury Eur J Pharmacol 2010 630 152 157 10.1016/j.ejphar.2009.12.025 20035747

[B26] Lundberg AH Granger N Russell J Callicutt S Gaber LW Kotb M Sabek O Gaber AO Temporal correlation of tumor necrosis factor-alpha release, upregulation of pulmonary ICAM-1 and VCAM-1, neutrophil sequestration, and lung injury in diet-induced pancreatitis J Gastrointest Surg 2000 4 248 257 10.1016/S1091-255X(00)80073-6 10769087

[B27] Pastor CM Morel DR Vonlaufen A Schiffer E Lescuyer P Frossard JL Delayed production of IL-18 in lungs and pancreas of rats with acute pancreatitis Pancreatology 2010 10 752 757 10.1159/000317283 21273803

[B28] Zhang XP Zhang J Ma ML Cai Y Xu RJ Xie Q Jiang XG Ye Q Pathological changes at early stage of multiple organ injury in a rat model of severe acute pancreatitis Hepatobiliary Pancreat Dis Int 2010 9 1 83 87 20133235

[B29] Qin X Dong W Sharpe SM Sheth SU Palange DC Rider T Jandacek R Tso P Deitch EA Role of lipase-generated free fatty acids in converting mesenteric lymph from a noncytotoxic to a cytotoxic fluid Am J Physiol Gastrointest Liver Physiol 2012 303 G969 G978 10.1152/ajpgi.00290.2012 22899820 PMC3469691

